# PET/MRI multimodality imaging to evaluate changes in glymphatic system function and biomarkers of Alzheimer’s disease

**DOI:** 10.1038/s41598-024-62806-5

**Published:** 2024-05-29

**Authors:** Hidehiko Okazawa, Munenobu Nogami, Shota Ishida, Akira Makino, Tetsuya Mori, Yasushi Kiyono, Masamichi Ikawa

**Affiliations:** 1 Biomedical Imaging Research Center, University of Fukui, 23-3, Matsuoka-Shimaizuki, Eiheiji-cho, Fukui, 910-1193 Japan; 2 Kyoto College of Medical Science, Nantan, Japan; 3 Department of Community Health Science, Faculty of Medical Sciences, University of Fukui, Fukui, Japan; 4 Department of Radiology, Kobe University Hospital, Kobe, Japan

**Keywords:** Glymphatic system, Amyloid imaging, PET/MRI, DTI-ALPS, Alzheimer’s disease, Alzheimer's disease, Molecular neuroscience

## Abstract

The glymphatic system is considered to play a pivotal role in the clearance of disease-causing proteins in neurodegenerative diseases. This study employed MR diffusion tensor imaging (DTI) to evaluate glymphatic system function and its correlation with brain amyloid accumulation levels measured using [^11^C]Pittsburgh compound-B (PiB) PET/MRI. Fifty-six patients with mild cognitive impairment and early Alzheimer’s disease (AD: 70 ± 11 y) underwent [^11^C]PiB PET/MRI to assess amyloid deposition and were compared with 27 age-matched cognitively normal volunteers (CN: 69 ± 10y). All participants were evaluated for cognitive function using the Mini Mental State Examination (MMSE) before [^11^C]PiB PET/MRI. DTI images were acquired during the PET/MRI scan with several other MR sequences. The DTI analysis along the perivascular space index (DTI-ALPS index) was calculated to estimate the functional activity of the glymphatic system. Centiloid scale was applied to quantify amyloid deposition levels from [^11^C]PiB PET images. All patients in the AD group showed positive [^11^C]PiB accumulation, whereas all CN participants were negative. ALPS-index for all subjects linearly correlated with PiB centiloid, MMSE scores, and hippocampal volume. The correlation between the ALPS-index and PiB accumulation was more pronounced than with any other biomarkers. These findings suggest that glymphatic system dysfunction is a significant factor in the early stages of Alzheimer’s disease.

## Introduction

The glymphatic system in the brain, recognized as a pivotal clearance mechanism, plays a crucial role in draining waste products from the brain parenchyma to the perivascular space through neurofluidic flow. Disturbances of the glymphatic system are assumed to induce deposition of waste products, which aggregate as disease proteins. Previous mouse experiments showed that the glymphatic system contributed to the clearance of β-amyloid and other detrimental proteins from the brain^1–3^. This is particularly significant in the context of Alzheimer's disease (AD), where the deposition of amyloid and tau is thought to play an important role in the process of pathologic progression prior to cognitive decline in AD patients^4^. If impairment of clearance mechanism causes such disease protein deposition, the glymphatic system dysfunction could be a major factor in the pathogenesis of AD as well as other neurodegenerative diseases. Monitoring and evaluating the glymphatic system function could, therefore, be crucial for both treatment and prediction of neurodegenerative pathologies.

The advent of amyloid and tau positron emission tomography (PET) imaging has led to a breakthrough in the differential diagnosis of dementia, particularly in diagnosing AD^5^. The molecular imaging technique is useful and widely used for the clinical diagnosis of neurodegenerative diseases by visually delineating pathological changes in the brain. The tracer accumulation in amyloid PET imaging reflects deposition of amyloid plaques and accumulation in tau imaging delineates neurofibrillary tangles caused by phosphorylated tau. Since disease protein deposition induces neurodegenerative changes, the relationship between impaired clearance mechanisms and the deposition of waste proteins is thus critical for understanding pathophysiologic changes in neurodegenerative diseases. Consequently, a combined analysis of functional changes in the glymphatic system with amyloid/tau deposition could unveil aspects of the neurodegenerative process in prodromal phase of AD. If there is a close relation between glymphatic system dysfunction and protein deposition, assessing glymphatic system function becomes significant for predicting deposition of waste products, and potentially preventing dementia and other neurodegenerative conditions, possibly leading to new therapeutic strategies.

Recently, the evaluation of glymphatic system function using magnetic resonance imaging (MRI) has gained attention, with several methods being proposed for this purpose. Taoka, et al. developed a novel index for estimating glymphatic system activity using diffusion tensor image (DTI) analysis along the perivascular space (ALPS) signals^6,7^. The ALPS-index allows for the evaluation of the glymphatic system without the need for a contrast agent. It is based on the principle that minimal perivascular water diffusion corresponds to an index close to one, with the index increasing as perivascular diffusivity increases. This approach has been utilized in various studies to investigate the relationship between glymphatic system activity and cognitive function, as well as tracer uptake of amyloid imaging in AD patients and cognitively normal (CN) participants^8–10^.

In the present study, [^11^C]methyl-2-(4′-methyl-aminophenyl)-6-hydroxybenzo-thiazole ([^11^C]PiB) PET/MRI was employed to obtain information on amyloid deposition in conjunction with other biomarkers from multiple MRI sequences. DTI data were acquired using two b-values of 1000 and 2000. In the previous studies, standardized uptake value (SUV) and its ratio to reference tissue (SUVR) were used at several brain regions for their analysis^9,10^. However, our study implements the centiloid scale as a measure for amyloid deposition in amyloid PET imaging^11^. The centiloid scale offers a comprehensive overview of amyloid deposition throughout the brain, which is more indicative to whole brain glymphatic system dysfunction.

## Material and methods

### Subjects

Fifty-six patients (28 men and 28 women, aged 70 ± 11 years), experiencing cognitive decline, diagnosed either with mild cognitive impairment (MCI) or early Alzheimer’s type dementia (DAT) were enrolled in the [^11^C]PiB PET/MRI study to evaluate cerebral amyloid deposition for assessment of pathophysiologic changes in dementia. Additionally, 27 age-matched healthy volunteers (11 men and 16 women, 69 ± 10 years) were also recruited to form the CN group. All participants were interviewed to evaluate their cognitive abilities using the Mini Mental State Examination (MMSE). [^11^C]PiB PET/MRI scans were conducted on all subjects to observe brain accumulation of PiB and to acquire MR images simultaneously. The study was approved by the Ethics Committee of the University of Fukui, Faculty of Medical Sciences (study protocol No. 20170225), adhering to its guidelines (Ethical Guidelines for Medical Science Research with Humans) and the Helsinki Declaration of 1975, as revised in 1983. Written informed consent was obtained from each participant.

### PET/MRI scanner

For PET and MRI data acquisition, we utilized a whole-body PET/MRI scanner (Signa PET/MR, ver. 26, GE Healthcare, Milwaukee, WI, USA)^12,13^. The scanner permits PET acquisition of 89 image slices in a 3D mode, with a slice thickness of 2.76 mm. Performance tests indicated that the intrinsic resolution of PET images to be 4.2–4.3 mm full width at half maximum (FWHM) in the transaxial direction. The PET/MRI scanner was calibrated beforehand using a dose-calibrator (CRC-12, Capintec Inc., NJ, USA) and a pool phantom with ^18^F-solution, according to the scanner manufacturer’s guidelines^12^.

### PET and MRI image acquisition and PET reconstruction

The patients underwent brain PET/MRI scans using a standard head coil (8-channel HD Brain, GE Healthcare), which facilitated simultaneous PET and MRI acquisition^12,14^. A 70-min list-mode 3D PET scan in time-of-flight (TOF) acquisition mode was started at the time of a bolus tracer injection of 700–750 MBq [^11^C]PiB via the antecubital vein. Concurrently, a 3D radial MR acquisition of the zero-echo time (ZTE) method in the axial direction was performed for PET data attenuation correction (AC) with the following parameters: field of view (FOV) 264 mm, a matrix 110 × 110 × 116, a voxel size 2.4 × 2.4 × 2.4 mm^3^, a flip angle 0.8°, number of excitations 4, bandwidth ± 62.5 kHz, and acquisition time of 41 s^14–16^. In the ZTE-AC method, the following process was used to create the MR-AC map based on a previous study^14^. Briefly, pre-filtering and histogram-based normalization were followed by intensity-based segmentation of the head, bias correction, identifying voxels affected by partial volume effects, and segmentation of the sinus, bone, and cavity masks. A pseudo-CT map was then generated from the ZTE intensity scaled linearly for bone tissue. This mapping was determined by fitting of registered CT and ZTE data in the bone density range. To convert the pseudo-CT into a MR-AC map, the images were re-sampled with a 60 × 60 × 25 cm^3^ FOV in a 128 × 128 × 89 matrix, and finally rescaled to 511 keV attenuation coefficients.

For evaluation of [^11^C]PiB accumulation, the static images from the 50–70 min timeframe of the dynamic list-mode PET data were used. Decay of PET data radioactivity was corrected to the starting point of each scan^17^. PET image reconstruction employed a 3D ordered-subset expectation maximization (OSEM) algorithm with the following parameter set: subset, 16; iteration, 2; transaxial post-gaussian filter cutoff of 4 mm in a 256 mm FOV with 2 × 2 mm^2^ pixel size. The SUV image, representing the average accumulation during this period, was calculated based on each patient’s body weight (BW: kg) and injection dose (ID: MBq) as follows: SUV = (PET count concentration [kBq/mL])/(ID/BW)^17^. To compare [^11^C]PiB accumulation in the brain, each SUV image was normalized by the individual SUV mean of the cerebellum and saved as the SUVR image.

High resolution 3D T1-weighted (T1WI) anatomical MRI was acquired in both axial and sagittal sections during the PET scan using the following parameters; repetition time = 8.5 ms; echo time = 3.2 ms; flip angle = 12°; FOV = 256 mm; 256 × 256 matrix; 136 slices; voxel dimension = 1.0 × 1.0 × 1.0 mm^3^^12,17^. Additional anatomical MR images such as T2-weighted (T2WI), FLAIR, etc., were acquired in the same position. Diffusion weighted images (DWI) for DTI-ALPS analysis were acquired during the PET scan using single-shot EPI (TE = Minimum; TR = 9327 ms; FOV = 240 mm; 240 × 240 matrix; pixel size = 1.9 × 1.9 mm^2^; 45 axial slices; slice thickness/gap = 3.0 mm/0 mm) with 30 distributed isotropic orientations for the diffusion-sensitizing gradients at b-values of 0, 1000 and 2000 s/mm^2^. DWI images were then converted to DTI images (b = 1000 and 2000) for further analysis of DTI-ALPS.

The volume of the hippocampal region was analyzed using the voxel-based specific regional analysis system for Alzheimer’s disease (VSRAD), a free software program, based on SPM and the standard Diffeomorphic Anatomical Registration Through Exponentiated Lie Algebra (DARTEL)^18^. Briefly, the gray matter (GM), white matter (WM), or cerebrospinal fluid (CSF) space images segmented from 3D-T1WI of each subject were anatomically standardized to a customized GM template, and then smoothed with an 8-mm FWHM isotropic Gaussian kernel. VSRAD provided regional *Z*-scores, defined as: ([control mean] − [individual value])/(control SD), to assess GM atrophy in each subject relative to a normal GM database.

### ALPS-index calculation

**Figure 1:**
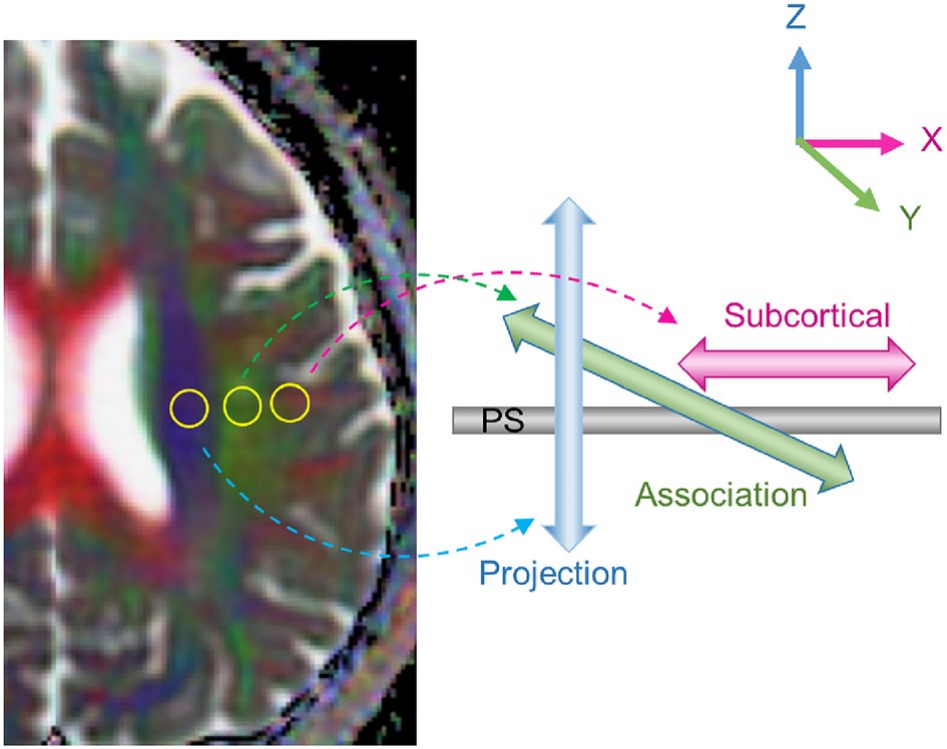
Schematic explanation of perivascular space (PS) diffusivity measurement using the DTI-ALPS method. Two regions of interest (ROIs) for calculation of ALPS-index were placed at the slice level of the upper lateral ventricles, in the projection (blue) and association (green) areas on DTI image. ROI value from subcortical area was not used for DTI-ALPS calculation because the subcortical neural fibers pass parallel to PS and the diffusivity would not reflect pure perivascular water diffusion. Direction of the neuron fibers at this level is defined as the x, y and z like the right upper index.

The ALPS-index was calculated to assess the glymphatic system activity based on the diffusivity of the projection and association fibers in individual participants, aligning with methodologies described in previous papers^6,7^. In briefe, diffusivity along the x-axis (D_x_), y-axis (D_y_), and the z-axis (D_z_) was measured as region of interest (ROI) values in three neural fiber areas: projection (D_xx__proj, D_yy__proj, D_zz__proj), the association (D_xx__assoc, D_yy__assoc, D_zz__assoc), and the subcortical (D_xx__subc, D_yy__subc, D_zz__subc), using diffusivity maps (Fig. 1). The diffusivity along the x-axis in the projection and association areas indicates water diffusion in the perivascular space (PS) without interference from neural fibers, reflecting glymphatic system activity. The diffusivity in the subcortical area would not reflect pure perivascular water diffusion due to the parallel orientation of subcortical neural fibers to the perivascular direction, which potentially obscures glymphatic diffusion^6^. The ALPS-index is provided as the ratio of two sets of diffusivity values perpendicular to the dominant fibers in the tissue, i.e., the ratio of mean of D_xx__proj and D_xx__assoc to the mean of D_yy__proj and D_zz__assoc;

$${\text{ALPS}}\;{\text{index}} = {\text{mean}}\;\left( {{\text{D}}_{{{\text{xx}}}} \_{\text{proj}},\;{\text{D}}_{{{\text{xx}}}} \_{\text{assoc}}} \right)/{\text{mean}}\;\left( {{\text{D}}_{{{\text{yy}}}} \_{\text{proj}},\;{\text{D}}_{{{\text{zz}}}} \_{\text{assoc}}} \right).$$

The ALPS-indexes, obtained from the ROI values in the upper paraventricular regions of both cerebral hemispheres (see Fig. 1), were averaged for each participant for further analysis.

### Centiloid scale calculation from amyloid PET

The centiloid scale is one of the standard quantitative measures in amyloid PET imaging. It evaluates amyloid deposition levels by normalizing outcomes across different analytical methods or PET ligands to a scale ranging from 0 to 100^11^. Its advantage lies in facilitating direct comparison of results across various institutes, regardless of the different PET tracers and analytical methods employed. Recently, several software packages have been developed to calculate centiloid scaling and are used for stage evaluation of patients with prodromal to early AD. To assess the level of [^11^C]PiB accumulation in the cerebral cortices, we used a software developed by Matsuda and Yamao in this study^19^, applying [^11^C]PiB SUVR and individual 3D-T1WI sagittal section images to obtain each participant’s global centiloid scale.

### Statistical analyses

Given that the PET/MRI scanner acquired images simultaneously, the location of PET and MRI images was identical, ensuring no head movement during the scan. The head position was verified through two anatomical MRI images at the beginning and end of PET acquisition. Statistical analysis was performed using SPSS (ver. 24), with a significance threshold set at *P* < 0.05. One-way analysis of variance (ANOVA) with a post-hoc Fisher’s LSD test was applied to examine differences in clinical variables and image values between the two groups. Pearson’s regression analysis was used to assess the linearity of correlations between clinical and image biomarkers.

To facilitate comparison with the amyloid deposition scale (PiB centiloid), DTI-ALPS, VSRAD Z-score, and MMSE score were normalized according to the following equations:

$${\text{nDTI}} - {\text{ALPS}} = \left( {{\text{ALPS}}_{{{\text{MAX}}}} {-}{\text{ALPS}}} \right)/\left( {{\text{ALPS}}_{{{\text{MAX}}}} - {1}} \right) \times {1}00$$

$${\text{nMMSE}} = \left( {{3}0{-}{\text{MMSE}}} \right)/{3}0 \times {1}00$$

$${\text{nVSRAD}} = \left( {{\text{VSRAD}}{-}{\text{VSRAD}}_{{{\text{MIN}}}} } \right)/{\text{VSRAD}}_{{{\text{MAX}}}} \times {1}00,$$

where nDTI-ALPS, nMMSE and nVSRAD represent the normalized values, with ‘MAX’ and ‘MIN’ denoting the maximum and minimum scores for all subjects, respectively.

## Results

All patients in the AD group, consisting of both MCI and early DAT, exhibited positive cortical [^11^C]PiB accumulation in the brain, while all participants in the CN group had negative accumulation. There were no differences in age range, mean age, and gender ratio between the AD and CN groups (Table 1). Notably, there was a significant difference in MMSE scores, indicating cognitive status, between the AD group (23.5 ± 3.8) and the CN group (27.8 ± 3.9) (*P* < 0.00001). The centiloid values obtained from [^11^C]PiB PET images also differed significantly between the AD (78.5 ± 33.4) and CN (0.4 ± 6.7) groups (*P* < 0.00001). The DTI-ALPS values for different diffusion sensitivity coefficients of b = 1000 and b = 2000 were both significantly lower in the AD group compared to the CN group (*P* < 0.00001 and *P* < 0.005, respectively).

**Table 1:** Participants demographics and biomarkers (mean ± SD).

	CN (n = 27)	AD (n = 56)	*P*-value
Age (years)	69.3 ± 9.6	69.9 ± 10.6	n.s. (0.80)
M:F	11:16	28:28	n.s. (0.43)
MMSE	27.8 ± 3.9	23.5 ± 3.8	< 0.00001
VSRAD (Z)	1.88 ± 0.92	1.20 ± 0.62	< 0.001
PiB Centiloid	0.4 ± 6.7	78.5 ± 33.4	< 0.00001
DTI-ALPS			
b = 1000	1.44 ± 0.09	1.29 ± 0.12	< 0.00001
b = 2000	1.38 ± 0.12	1.28 ± 0.13	< 0.005

CN, cognitively normal control; AD, Alzheimer’s disease; MMSE, mini-mental state examination; VSRAD, voxel-based specific regional analysis system for Alzheimer’s disease; DTI-ALPS, diffusion tensor image analysis along the perivascular space signals; n.s., not siginificant.

**Figure 2:**
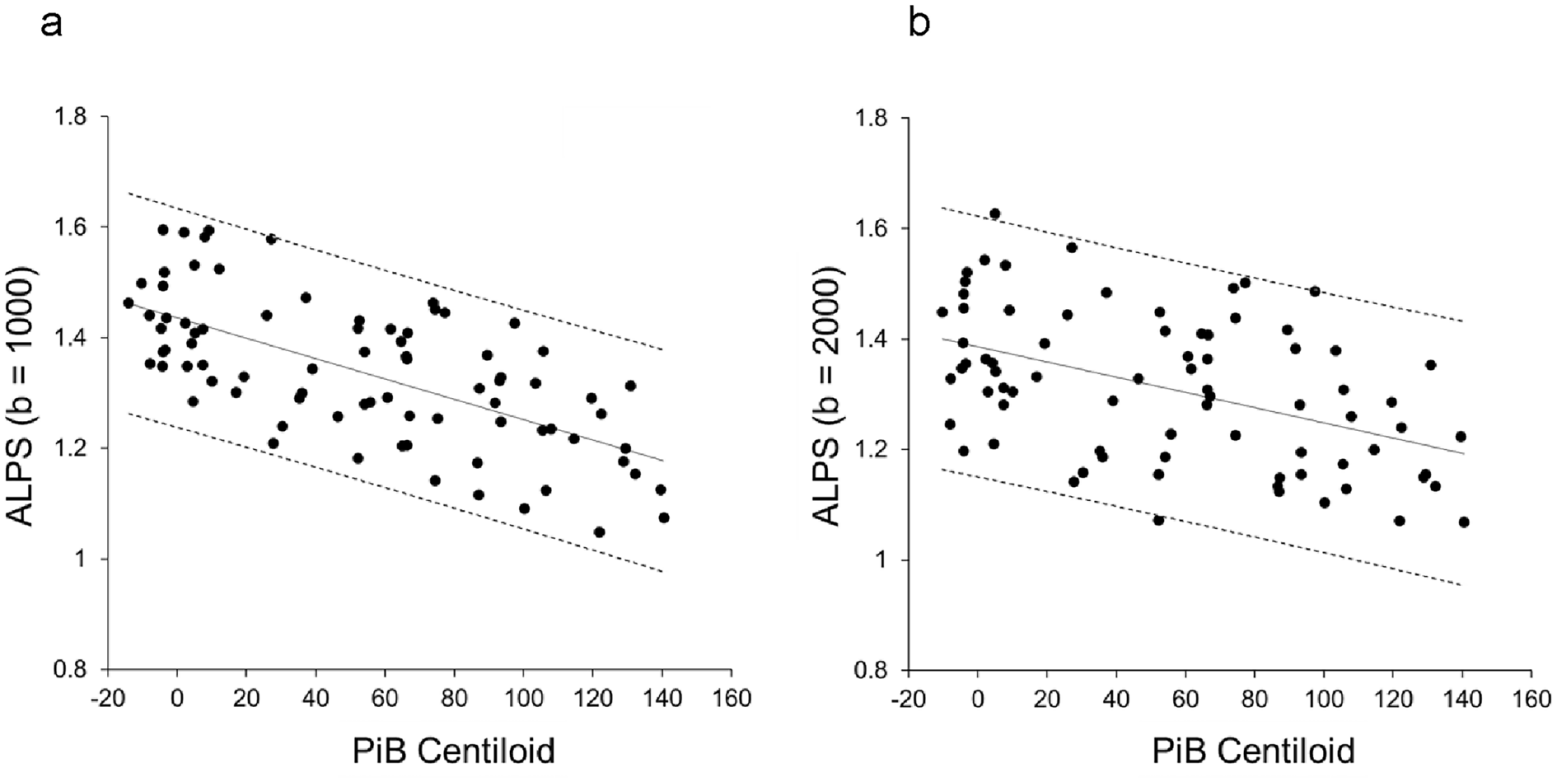
Relationship between PiB centiloid and DTI-ALPS calculated from b = 1000 (**a**) and b = 2000 (**b**) DTI images for all subjects. Both ALPS-indexes correlated well with PiB centiloid scores (*P* < 0.00001). The ALPS-index for b = 1000 showed a better correlation coefficient (r = 0.65) compared to b = 2000 (r = 0.47), and the narrower confidence interval for b = 1000 indicates smaller variance. Solid lines are linear regression lines and dashed lines indicate confidence intervals.

**Figure 3:**
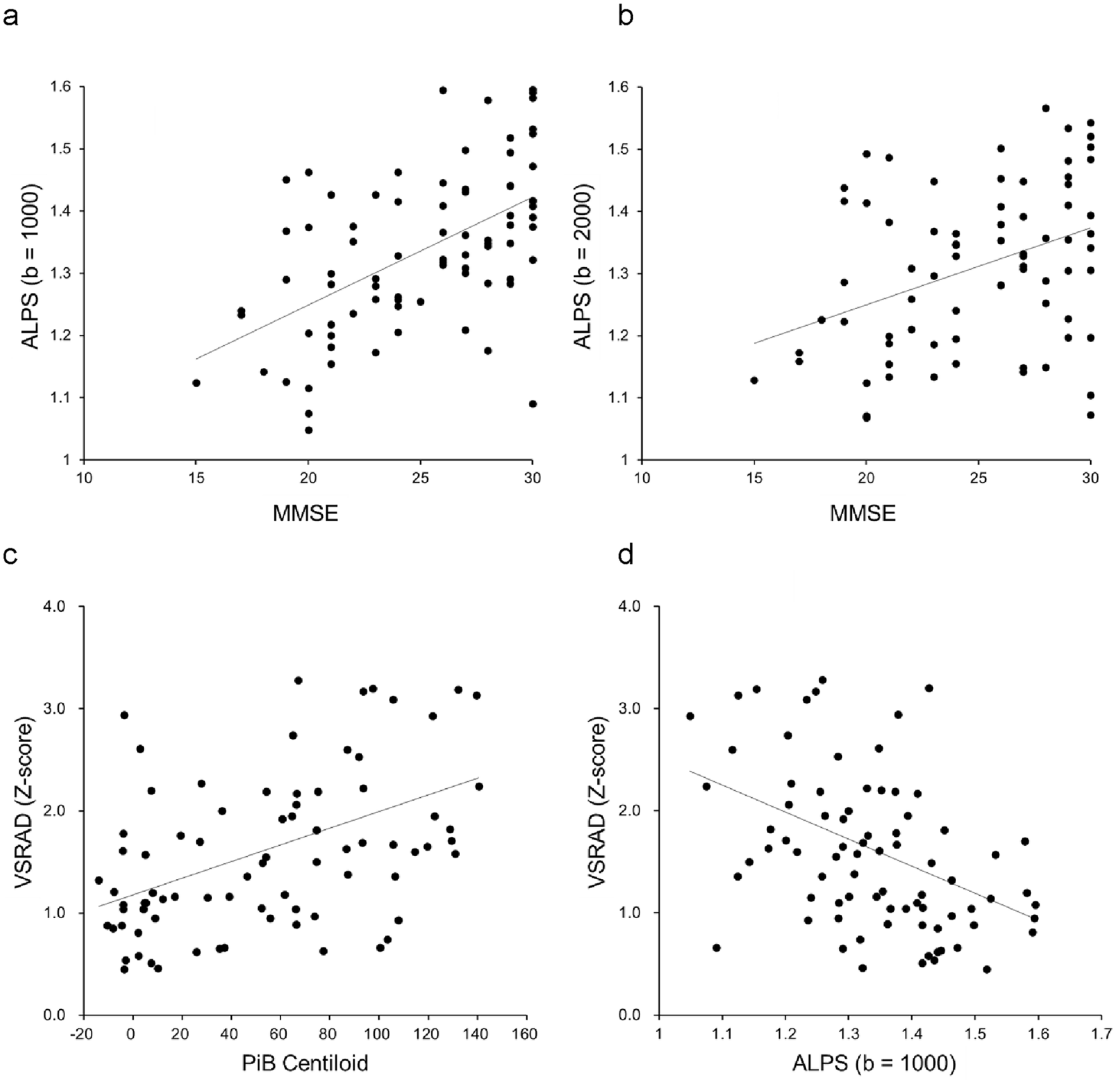
Representative correlations between ALPS-index, MMSE and VSRAD Z-score. MMSE showed significant correlations with both DTI-ALPS, with a more robust linear correlation observed for b = 1000 (**a**: r = 0.55, *P* < 0.00001) than for b = 2000 (**b**: r = 0.37, *P* < 0.001). VSRAD Z-score was also significantly correlated with PiB centiloid (**c**: r = 0.44, *P* < 0.00001) and DTI-ALPS (**d**: r = 0.40, *P* < 0.001). Lines are linear regression lines.

Figure 2 shows the relationship between PiB centiloid and DTI-ALPS across all participants. DTI images acquired with b = 1000 and b = 2000 provided ALPS-indexes that showed a strong correlation with PiB centiloid scores. While both DTI-ALPS indexes demonstrated significant linear correlations, DTI-ALPS with b = 1000 showed a higher correlation coefficient (r = 0.65) compared to b = 2000 (r = 0.47), with smaller variance, observed in a narrower confidence interval, for b = 1000. Cognitive function, as measured by MMSE, also correlated significantly with DTI-ALPS, with a more robust linear correlation observed for b = 1000 (r = 0.55, *P* < 0.00001) than for b = 2000(r = 0.37, *P* < 0.001) (Fig. 3a,b). The Z-scores of hippocampal volume, calculated using VSRAD software, also displayed significant correlations with both PiB centiloid and DTI-ALPS (Fig. 3c,d). Table 2 summarizes the slopes, intercepts, and correlation coefficients for all comparisons.

**Table 2:** Results of Pearson’s regression analysis.

	ALPS (b = 1000)				ALPS (b = 2000)			
	Slope	Intercept	r	*P*	Slope	Intercept	r	*P*
MMSE	0.017	0.902	0.55	< 0.00001	0.012	1.002	0.37	< 0.001
PiB Centiloid	− 0.0018	1.436	0.65	< 0.00001	− 0.0014	1.386	0.47	< 0.00001
VSRAD (Z)	− 2.65	5.16	0.40	< 0.001	− 1.41	3.47	0.22	< 0.001

MMSE, Mini Mental State Examination; ALPS, analysis along the perivascular space signals; VSRAD, voxel-based specific regional analysis system for Alzheimer’s disease.

**Figure 4:**
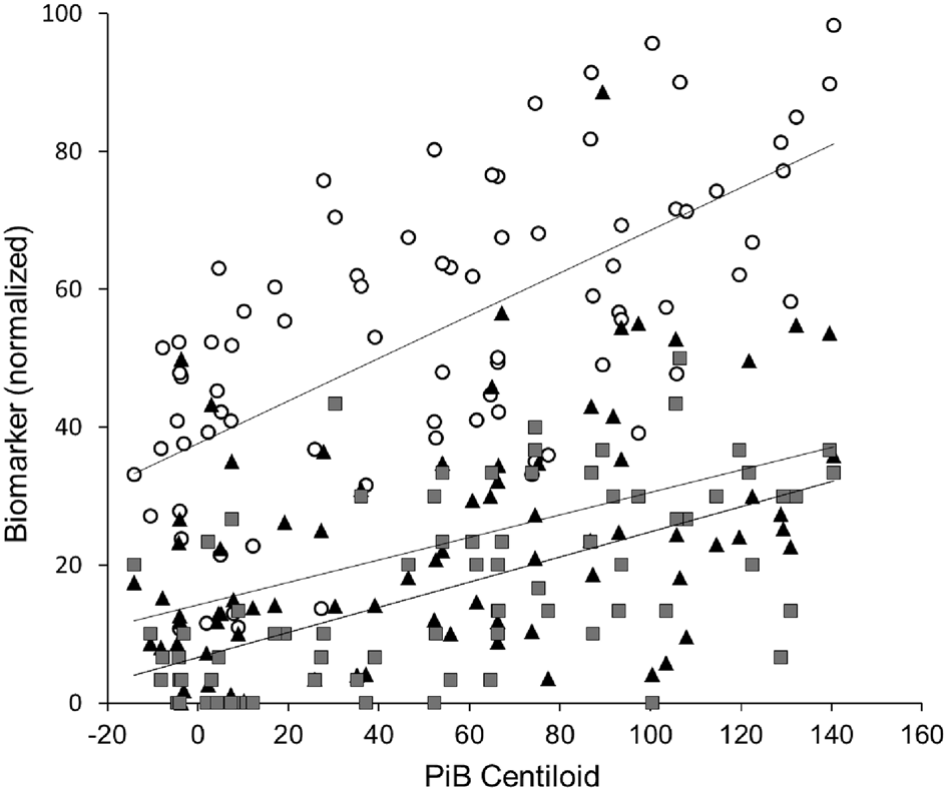
DTI-ALPS, VSRAD Z-score and MMSE score were compared with PiB centiloid after normalization. nDTI-ALPS (○) had a speeper slope and a better correlation coefficient (0.31, r = 0.65) compared to nVSRAD (▲, 0.16, r = 0.43) and nMMSE (■, 0.18, r = 0.61). Lines are linear regression lines for each pair of correlation.

In examining changes in each index relative to the progression stages of AD, as scaled by amyloid deposition, DTI-ALPS (b = 1000), VSRAD Z-score, and MMSE score were normalized to compare with PiB centiloid (Fig. 4). The normalized DTI-ALPS (nDTI-ALPS) showed a steeper slope of linear correlation (0.31, r = 0.65) with PiB centiloid than both normalized VSRAD (nVSRAD) (0.16, r = 0.43) and normalized MMSE (nMMSE) (0.18, r = 0.61), suggesting that DTI-ALPS correlates more closely with amyloid deposition than with changes in hippocampal volume or cognitive dysfunction.

## Discussion

In this study, we explored the relationship between glymphatic system function, as measured by DTI-ALPS, and other biomarkers commonly used for assessment of AD progression, i.e. amyloid deposition, hippocampal atrophy, and cognitive function. All biomarkers demonstrated a significant linear correlation with the ALPS-index, indicating a close relationship between changes in glymphatic system function in the brain and the neurodegenerative changes attributed to deposition of disease protein. In comparison, the strongest correlation was observed between the ALPS-index and amyloid deposition, particularly in terms of the correlation coefficient. The slope of the regression line between PiB centiloid and normalized biomarkers was steeper for nDTI-ALPS compared to other biomarkers, suggesting that glymphatic system dysfunction is intricately linked to amyloid deposition, which precedes other functional declines caused by drainage dysfunction. Hippocampal volume, as assessed by VSRAD, and cognitive function, evaluated by MMSE, showed a weaker correlation with the ALPS-index, implying that glymphatic system dysfunction and amyloid deposition might precede brain atrophy and cognitive decline in neurodegenerative changes.

Previous studies have compared DTI-ALPS with amyloid deposition using SUVR values for PET tracer accumulation. These studies showed that ALPS-index correlated with SUVR in specific cerebral regions in AD patients and CN subjects. Park, et al. found a significant linear correlation between DTI-ALPS and [^18^F]flutemetamol SUVR in the paracentral region of 123 elderly subjects (r = 0.218, *P* = 0.016), but not in other brain regions^9^. Hsu, et al. examined the relationship between ALPS-index and [^18^F]AV-45 SUVR across various brain regions in 50 participants, finding significant correlations except in the parahippocampal region, though the correlation coefficients were modest^10^. Our study used PiB centiloid calculated from whole brain PET images to quantify the total amount of cortical amyloid deposition in each subject. The centiloid scale, a common parameter for assessing amyloid deposition regardless of amyloid PET tracer types, provides global deposition levels of amyloid. Considering that glymphatic system dysfunction is observed as a global change in animal studies^20^, comparing global changes in amyloid deposition appears appropriate to observe relationship between clearance and deposition of disease proteins.

In this study, DTI-ALPS indexes were obtained from two MR sequences with b = 1000 and 2000. Both sets of DTI images showed significant correlations with other biomarkers; however, the correlation was better with DTI from b = 1000, both in terms of the correlation coefficient and the range of variance. DTI with b = 1000 is commonly used for assessing white matter function of neural connectivity due to its superior image quality. The previous study by Taoka, et al. comparing DTI-ALPS and MMSE scores also showed similar results to ours with a better correlation for DTI with b = 1000 compared to b = 2000^6^. Therefore, DTI images with b = 1000 are considered more suitable for calculating the ALPS-index and assessing glymphatic system function.

Various biomarkers have been examined in relation to AD stage progression^21^. Recent studies focusing on tau deposition for AD staging have shown that the in vivo pathologic staging correlates well with other biomarkers such as CSF findings, cognitive function, and GM volume^22^. These studies suggest that biomarker changes begin with amyloid levels in the CSF and brain, followed by changes in tau in the CSF and brain, cerebral volume reduction, memory disturbances, and other clinical symptoms. This aligns with prior hypotheses^4,5^. Additional studies have incorporated biomarkers of neuroinflammation such as microglial or glial activation and oxidative stress^23–26^, which are linked to early pathological changes in AD biomarkers. Our findings suggest that the glymphatic drainage system is also closely related to initial amyloid deposition. While the causal relationship between impaired clearance and amyloid deposition remains unclear, glymphatic system dysfunction appears to be a crucial factor in the neurodegenerative changes observed in dementia.

## Conclusion

The function of the glymphatic system, as measured by DTI-ALPS, shows a strong correlation with amyloid deposition in the early stages of AD. These findings suggest that both factors are pivotal in inducing neurodegenerative changes and cognitive dysfunction.

## Data Availability

The datasets used and/or analyzed during the current study are available from the corresponding author on reasonable request.
